# Perceptions of football players regarding injury risk factors and prevention strategies

**DOI:** 10.1371/journal.pone.0176829

**Published:** 2017-05-01

**Authors:** Astrid Zech, Kai Wellmann

**Affiliations:** 1 Institute of Sports Science, University of Jena, Jena, Germany; 2 Institute of Human Movement Science, University of Hamburg, Hamburg, Germany; Instituto Cajal-CSIC, SPAIN

## Abstract

Current approaches regarding injury prevention focus on the transfer of evidence into daily practice. One promising approach is to influence attitudes and beliefs of players. The objective of this study was to record player’s perceptions on injury prevention. A survey was performed among players of one German high-level football (soccer) club. 139 professional and youth players between age 13 and 35 years completed a standardized questionnaire (response rate = 98%). It included categories with (1) history of lower extremity injuries, (2) perceptions regarding risk factors and (3) regularly used prevention strategies.

The majority of players (84.2%) had a previous injury. 47.5% of respondents believe that contact with other players is a risk factor, followed by fatigue (38.1%) and environmental factors (25.9%). The relevance of previous injuries as a risk factor is differently perceived between injured (25%) and uninjured players (0.0%). Nearly all players (91.5%) perform stretching to prevent injuries, followed by neuromuscular warm up exercises (54.0%). Taping is used by 40.2% of previously injured players and 13.6% of players without a history of injuries. In conclusion, the perception of risk factors and performed preventive strategies are inconsistent with scientific evidence. Future transfer strategies should incorporate the players beliefs and attitudes.

## Introduction

Playing football (soccer) on a recreational or professional performance level is associated with an increased risk of injury. Compared to other sports, there is a relatively high number of injuries per football game [1, 2] as well as one of the highest proportion of injured players during a tournament [3]. The majority of injuries are non-contact and located at lower extremities. Numerous studies also indicate that injury rates in football vary depending on performance / competition levels, sex and age [4–6]. During international tournaments such as the FIFA world cup, players seem to be at higher risk for injuries [7, 8] than during national leagues seasons [9]. Reported intrinsic risk factors for lower extremity injuries are a previous injury [10], increased range of motion [4, 11, 12], limited postural control [11] and fatigue [12, 13].

Since lower extremity injuries often require longer medical treatment periods and result in time loss or impaired function during playing [14], special attention has been given on the development and validation of effective preventive strategies during the last 15 years. Evidence exists on the benefits of bracing, taping and neuromuscular exercising for preventing injuries during team sports [15, 16]. However, although it has been already shown that injury prevention strategies can be successfully promoted among team coaches and physiotherapists [17, 18] it is still unknown whether and which of these effective measures are generally accepted and regularly used in professional and youth football. The compliance of the athlete with the preventive measure seems to be a key factor for the successful implementation of effective strategies in sports practice [19, 20]. Soligard et al. [20] showed that players with high compliance rates had a significantly lower injury risk than players with low compliance rates. Therefore, it seems problematic that various studies or reviews reported a poor compliance with injury prevention measures such as neuromuscular training or regular bracing [16, 21–23].

As a consequence, a primary focus of future injury prevention strategies should be on improving compliance among the athletes to truly effective measures during competition and football practice. One promising approach is to gain knowledge regarding the player’s beliefs and attitudes towards injury prevention [19, 24]. According to the current theoretical framework for injury prevention, successful measures should aim for the modification of the players’ behavior and attitude towards risk factors and preventive measures [19, 25]. However, to our knowledge, no study previously collected data on individual beliefs and adopted strategies of injury prevention among football athletes. It is therefore unknown whether and to what extent evidence-based measures have been already adopted by professional or recreational players.

The objective of this study was to collect first data on perceptions on injury mechanisms, risk factors and preventive measures as well as effectively used strategies during practice and competition among professional and youth football players.

## Methods

### Study design

The study, a cross-sectional survey of among professional and youth football players in one German high-level football club, conducted in June 2015 was approved by the Ethics Committee of the Faculty of Social and Behavioural Sciences of the University of Jena (protocol no. FSV 15/14) and followed the principles of the Helsinki Declaration.

### Participants, recruitment and setting

Active players of the professional team (Bundesliga, second division during the time of the survey) and youth teams (U-23, U-19, U-17, U-16 and U-15) of one German football club were asked to participate. Although there were also U-12, U-13 and U-14 teams in the participating football club, inclusion was restricted to the U-15 due to the experience with injuries and preventive measures. No exclusion criteria were defined regarding injury status, playing positions, or performance level. The anonymous survey was conducted before the start of preseason practice by a researcher not involved in the training routines or medical practice. The questionnaire was distributed and collected by the researcher on the same day. A statement at the title page of the questionnaire included information regarding the study purpose and the anonymity of responses. The response to the survey was assumed to indicate the informed consent of the athletes. Due to the fact that minors were involved, parental consent for participation was obtained by the coaches.

### Procedure

The questionnaire was developed according to the current evidence of injury prevention [11, 12, 15, 16, 23] as well as established theoretical models [24–27]. It included three categories with 10 questions to be answered. In agreement with the stages of the injury sequence model of van Mechelen [24] we collected data on the injury history, perceived risk factors, and believes and practices regarding preventive measures. Question generation was based on a literature review and valuable discussions with experts. Questions were presented in a language appropriate for athletes in the adolescence and young adult age. Each category of interest had single questions with a dichotomous response (yes or no). The questionnaire was pilot-tested in three local non-professional football teams. Results from these teams indicated that the majority of participants understood the questions and response options. In the first part, data on players’ characteristics (age, height, weight and team) were collected. The second part contained questions on history of injuries. Participants were asked to report each injury of the foot, ankle, knee, hip, thigh and groin that ever occurred during football practice or game with regard to the year, kind, leg side, estimated time loss and circumstances (with or without contact, game or practice). The time loss was divided into the following categories: 1–6 days (minor), 7–27 days (moderate) and more than 28 days (severe). In the third part, participants were asked (a) to estimate the influence of intrinsic (physical condition, coordination, muscle impairments, fatigue, diet, previous injury, attentiveness) and extrinsic factors (other player/contact, equipment / environment, climatic conditions) on lower extremity football injuries; (b) to rate the importance of injury prevention from one (highest priority) to five (lowest priority) as well whether they feel adequately informed about injury prevention; and (c) to report which of the following prevention strategies they regularly use for injury prevention: stretching, specific warm-up exercises, specific strength exercises, bracing, taping, shoe insoles, face masks, medical corsets.

### Data analysis and statistics

Descriptive statistics were used to evaluate demographic data. Continuous variables were reported as mean and standard deviation and categorical variables were presented as percentages of yes answers (%). Personal opinions on the importance of injury prevention were assessed on five-point Likert scales (i don’t care, not important, somehow important, important, and very important).

Pearson’s Chi-Square test was used to test whether these injury rates, perceptions on potential risk factors and preventive measures were differently distributed between teams (of different age) or between injured and non-injured players. The significance level was set to 5%. SPSS version 21.0 for Windows (SPSS, Chicago, Illinois, USA) was used for statistical analysis.

## Results

Out of 142 eligible athletes in six teams, 139 players returned the questionnaire (response rate = 98%). Players not returning questionnaires were from the first team (n = 2) as well as the U19 team (n = 1). Overall, 24 professional and 115 youth players participated in this survey. All returned questionnaires were analyzed. The age of respondents ranged between 13 and 35 years. A detailed description of the participants is given in Table 1. Teams differed significantly (p < .001) regarding age, mass and height.

**Table 1:** Players characteristics for professional and youth teams of one football club and p-values for differences between teams.

	First Team (Adults)	U-23	U-19	U-17	U-16	U-15	Team differences (p-value)
	24	18	25	17	20	35	
**Mean age (SD) in years**	24.9 (4.0)	21.0 (3.7)	17.1 (0.8)	15.8 (0.4)	14.8 (0.5)	13.6 (0.5)	< .001
**Mean height (SD) in cm**	184.1 (6.9)	179.8 (6.3)	178.1 (6.3)	181.2 (5.3)	178.1 (7.8)	168.0 (10.0)	< .001
**Mean body mass (SD) in kg**	80.0 (8.2)	75.8 (6.8)	71.7 (5.2)	70.8 (9.2)	66.7 (8.4)	55.3 (9.2)	< .001
**Previous lower extremity injury (% yes)**	100.0	72.2	84,4	82,4	90.0	77,1	.141
**Mean number of injuries per player (SD)**	2.8 (1.7)	1.9 (1.9)	1.8 (1.3)	2.5 (2.4)	1.8 (1.0)	1.1 (0.8)	.002

### History of injuries

84.2 percent of respondents reported a previous injury in the lower extremities. The percentage of previously injured players ranged between 100% (first team) and 72.2% (U23) (p = .141). Significant differences between teams were found regarding the rate of injuries per player (p = .002). Most injuries of players between U17 and the first team occurred at the ankle joint whereas the younger teams (U16 and U15) reported more injuries at the thigh / groin. The rate of players with ankle injuries was highest in the first and lowest in the youngest team (p = .006) (Table 2). More than two third of professional players experienced an injury at the ankle joint. Significant team differences (p = .026) were found regarding the severity of ankle injuries. 45.8% of first team players and 8.6% of U15 players reported injuries with time loss of more than 28 days. No differences between teams were found for knee (p = .662) as well as thigh and groin injuries (p = .738).

**Table 2:** Percentage of injured players and Pearson-Chi-Square test p-values for differences between teams.

		Ankle			Knee			Thigh / Groin		
	n	Previous injury (% yes)	Time loss >28d (% yes)	Without contact (% yes)	Previous injury (% yes)	Time loss >28d (% yes)	Without contact (% yes)	Previous injury (% yes)	Time loss >28d (% yes)	Without contact (% yes)
**First Team** **(Adults)**	24	75.0	45.8	45.8	41.7	29.2	16.7	62.5	20.8	45.8
**U23**	18	55.6	33.3	44.4	38.9	33.3	27.8	50.0	5.6	38.9
**U19**	25	60.0	16.0	44.0	48.0	20.8	40.0	52.0	24.0	40.0
**U17**	17	58.8	29.4	52.9	58.8	17.6	41.2	58.8	17.6	41.2
**U16**	20	45.0	30.0	50.0	45.0	20.0	35.0	70.0	25.0	55.0
**U15**	35	25.7	8.6	28.6	34.3	14.3	25.7	51.4	17.1	45.7
**all**	139	51.1	25.2	42.4	43.2	21.7	30.2	56.8	18.7	44.6
**Team differences (p-value)**		.006	.026	.527	.662	.615	.441	.738	.674	.920

### Perceptions on injury risk factors

Out of all respondents, 47.5% believed that physical contact with other players is a risk factor for lower extremity injuries, followed by physical fatigue (38.1%), environmental factors (including equipment, 25.9%), muscle impairments (25.7%) and previous injuries (21.7%) (Table 3). Only few players considered physical condition deficits (2.2%), diet (3.5%), climatic conditions (5.0%) and coordination deficits (7.2%) important for the lower extremity injury risk. Significant differences between athletes with and without previous injuries were found for answers regarding the influence of fatigue (p = .036), previous injuries (p = .007) and environment (.003). All three factors were rated higher by previously injured players than by those without injuries. Except for environmental factors (p = .002), teams generally agreed in their perceptions on potential risk factors for injuries.

**Table 3:** Player’s perceptions on potential risk factors for injuries (% yes answers) and Pearson-Chi-Square test p-values for differences in “yes” answers between teams as well as players with and without previous injuries.

	First Team (Adults)	U23		U19	U17	U16	U15	all	With previous injury	Without previous injury	Team differences (p-value)	Previous injury differences (p-value)
n	24	18		25	17	20	35	139	117	22		
**Intrinsic Risk Factors**												
**Physical condition**	4.2	0.0	0.0		11.8	0.0	2.9	2.2	2.6	0.0	.074	. 448
**Coordination**	12.5	16.7	0.0		5.9	15.0	0.0	7.2	6.8	9.1	.077	.707
**Muscle impairments**	25.0	11.1	36.0		29.4	25.0	22.9	25.2	27.4	13.6	.592	.174
**Fatigue**	45.8	38.9	32.0		52.9	45.0	25.7	38.1	41.9	18.1	.383	.036
**Diet**	8.0	0.0	0.0		11.8	5.3	0.0	3.5	7.7	0.0	.956	.179
**Previous Injury**	25.0	27.8	32.0		29.4	15.0	5.9	21.7	25.0	0.0	.257	.007
**Attentiveness**	4.2	5.6	8.0		23.5	5.0	14.3	10.1	10.3	9.1	.292	.868
**Extrinsic Risk Factors**												
**Other Player (Contact)**	58.3	50.0		52.0	58.8	45.0	31.4	47.5	50.4	31.8	.313	.109
**Equipment / Environment**	20.8	16.7		44.0	52.9	30.0	5.7	25.9	30.8	0.0	.002	.003
**Climatic Conditions**	0.0	11.1		4.0	0.0	10.0	5.7	5.0	6.0	0.0	.456	.239

### Injury prevention strategies

The majority of respondents consider injury prevention important or very important (Table 4). Answers differed not between teams or players of different injury status. Most players feel that there are adequately informed about effective injury prevention strategies although the rate differed significantly (p = .029) between teams. 91.5% of all players declared regularly to use stretching exercises in order to prevent injuries. Rates were not different between teams and injured vs. non-injured players. About half of all respondents (54.0%) perform specific warm up exercises and one third (33.1%) specific strength exercises (Table 5). External measures used regularly by players are taping (27.3%), shoe insoles (27.0%) and bracing (11.5%). While agreement in answers was found between teams, the injury status significantly influences the prevalence of taping (p = .017) and shoe insoles (p = .009) use. Both measures are more frequently used by players with previous injuries. Nevertheless, some players that never experienced lower extremity injuries reported regularly to use taping (13.6%) and bracing (9.1%).

**Table 4:** Importance of injury prevention (% yes answers).

	n	I don’t care	Not important	Somehow important	important	Very important
**First Team** **(Adults)**	24	0.0	0.0	0.0	29.2	70.8
**U23**	18	0.0	0.0	0.0	22.2	77.8
**U19**	25	4.2	4.2	4.2	20.8	66.7
**U17**	17	0.0	0.0	0.0	41.2	58.8
**U16**	20	0.0	0.0	5.0	35.0	60.0
**U15**	35	0.0	0.0	2.9	31.4	65.7
**all**	139	0.7	0.7	2.2	29.5	66.2
**With previous Injury**	117	0.9	0.0	2.6	29.5	67.2
**Without previous Injury**	22	0.0	4.5	0.0	31.8	63.6
**Team differences (p-value)**	.795					
**Previous injury differences (p-value)**	.191					

**Table 5:** Self-reported prevention strategies for injury prevention during football practice and / or competition (% yes answers).

	First Team (Adults)	U23	U19	U17	U16	U15	all	With previous injury	Without previous injury	Team differences (p-value)	Previous injury differences (p-value)
n	24	18	25	17	20	35	139	117	22		
**Do you feel adequately informed about injury prevention?**	91.7	61.1	83.3	82.4	75.0	82.4	80.4	80.2	81.8	.029	.825
**Which of the following prevention strategies do you use regularly?**											
**Stretching**	95.8	88.9	88.0	94.1	90.0	91.4	91.4	91.5	90.0	.933	.934
**Specific Warm-up Exercises**	54.2	44.4	56.0	82.4	50.0	45.7	54.0	53.8	54.5	.199	.952
**Specific Strength Exercises**	33.3	33.3	56.0	23.5	35.0	20.0	33.1	34.2	27.3	.095	.527
**Bracing**	0.0	11.1	16.0	29.4	5.0	11.4	11.5	12.0	9.1	.081	.698
**Taping**	33.3	33.3	44.0	58.8	30.0	25.7	27.3	40.2	13.6	.253	.017
**Shoe Insoles**	25.0	16.7	28.0	41.2	50.0	14.3	27.0	31.6	4.5	.053	.009
**Face Masks**	0.0	0.0	0.0	0.0	0.0	0.0	0.0	0.0	0.0	-	-
**Medical Corsets**	0.0	0.0	0.0	0.0	0.0	2.9	0.7	0.9	0.0	.701	.663

## Discussion

This survey presents data on injury rates and current perceptions regarding injury risk factors as well as information regarding prevention strategies used in football. Although there is general agreement that the player’s beliefs and attitudes towards injury prevention are a key factor for the successful implementation of effective measures [19, 24], this is, to our best knowledge the first survey among professional and youth athletes regarding the players individual injury prevention behavior. The questionnaire categories were developed according to the ‘sequence of injury prevention’ proposed by van Mechelen et al. [24]. It describes subsequent stages starting with the relevance of the problem in terms of incidence followed by risk factor identification and the use as well as assessment of preventive measures. Answers on specific risk factors and preventive strategies were built upon current evidence [11, 12, 15, 16, 23], theoretical models [26–29] and expert beliefs. However, while pilot tested, discussed with experts and adjusted accordingly, the developed questionnaire was used for the first time in this study. Therefore, we cannot fully exclude potential confounding factors and results should be considered hypothesis-generating.

The self-reported history of lower extremity injuries among respondents showed that at least two-thirds of football players of age 13 and above previously experienced an acute injury during practice or play. The number of injuries per player was lowest in the youngest team and highest in the professional team. This can be probably explained by the higher number of years of playing football as well as the higher risk in professional sports [9]. However, due to the retrospective questionnaire, the reported data of lower extremity injuries should be viewed cautiously. Several authors [30, 31] showed that retrospective surveys considerably underestimate the true incidence of sports injuries compared to prospective study designs or a mix of both methods. The true number of players with previous injuries as well as the injury incidence per player is therefore probably higher than reported in this study.

Perceptions and individual beliefs regarding lower extremity injury risk factors were similarly distributed among teams as well as between players with and without an injury history. Respondents generally believed that multiple intrinsic and extrinsic factors could be responsible for the occurrence of an injury during practice or play. Almost half of all athletes with injury history consider the physical contact with other players as a potential risk factor whereas only one-third of self-reported non-injured athletes answered positively in this item. Injury data of major tournaments such as the world cup showed an incidence of non-contact injuries between 35 and 45 percent [7]. Higher prevalence of non-contact injuries (about 50 percent) was reported for less skilled and youth players [32, 33]. Furthermore, severe acute injuries of lower extremities in football seem to be more often caused by non-contact than by contact with other players [34]. Hence, in our study, players with a history of injuries slightly overestimate the influence of contact on lower extremity injuries in football.

Frequently perceived intrinsic risk factors among respondents are physical fatigue (38%) and muscle impairments (25%). Muscle deficits have indeed been shown to be positively associated with the occurrence of hamstring strain [35] and groin injuries [36] during sport. While physical fatigue is a widely suggested risk factor for football sport injuries [12] no study ever investigated the direct influence of exhaustion during a game and the occurrence of injury. However, Frisch et al. [13] showed that the general perceived physical fatigue at preseason is a risk factor for injuries in youth football during a season. It may also be possible that the increased injury prevalence with increasing time of play [7, 8, 37] is at least partly a result of physical exhaustion. Other studies showed impairments of postural and neuromuscular control after different fatigue protocols [38] which were particularly evident in players with previous lower extremity injuries [39]. However, although numerous studies have shown an association between lower extremity injuries and neuromuscular / postural control impairments only few respondents in our studies considered coordination deficits as a risk factor for injuries in football.

Nearly all respondents declared injury prevention important or very important and most of them reported to feel well informed regarding this topic. The injury prevention strategies provided in the questionnaire included a range of external/assistive (e.g. taping, bracing) and internal/active prophylactic measures (e.g. exercises) for which either evidence exists [15, 16] and/or that were regularly used according to expert opinions (coaches, athletic trainer, physiotherapists, player). Almost all respondents declared to use regular stretching for injury preventions. However, no evidence exists on any benefits of passive or active stretching regarding the prevention of injuries in sports [40, 41]. Moreover, acute stretching before practice or play seem to have no effect or is even contradictory for a subsequent strength, jump or fast running performance [42]. In spite of this, stretching is apparently still the intervention of choice among players and team physicians [43] when aiming for injury prevention. This emphasizes the urgent need of effective transfer of evidence based knowledge on effective prophylactic measures to real football practice. About a half of all players reported regularly to perform specific warm up exercises in order to prevent injuries in football indicating an adequate acceptance of this measure among active players. There exists comprehensive evidence on the prophylactic benefits of neuromuscular warm up exercises including balance, plyometric and strength exercises [15, 16]. As expected, significant differences in answers between players with and without injury history were found regarding the use of passive prophylactic measures. A previous injury is a major risk factor for a recurrent injury at lower extremities [10]. While only one out of four of previously injured players in this survey considered a previous injury as potential risk factor about 40 percent of them reported regularly to use taping as a prophylactic measure. The rate was considerably lower among respondents without a history of lower extremity injuries. To date, only few studies investigated the effects of taping on the incidence of sports injuries. Nevertheless, there is a tendency towards its effectiveness for the prevention of ankle sprains [15]. Although more evidence exists for the benefits of bracing [23, 41] only 12 percent of respondents use this measure for prophylactic reasons.

The greatest limitation of this survey is the retrospective study design which majorly increase the bias regarding the assessment of true injury history. Other limitations include the use of team players of only one football club. Whereas this ensured homogeneity of participants it similarly reduces the generalizability of findings to players of other clubs or leagues. Future surveys across different regions are therefore needed to generate a comprehensive overview regarding performed strategies and the need of transfer of scientific findings to practice to improve the regular use and individual acceptance of truly effective measures.

## Conclusion

In conclusion, the perceived risk factors and used preventive strategies are mostly consistent among teams of different age and professional status. The athletes generally believed that multiple intrinsic and extrinsic factors could be responsible for the occurrence of lower extremity injuries, including contact, physical fatigue and muscle impairments. Only few respondents considered neuromuscular and postural control impairments as major risk factor for injuries. Nearly all athletes perform stretching before a match or practice in order to prevent injuries although no scientific evidence exists for this measure. Other frequently performed prevention strategies are specific warm up exercises and taping / bracing. The results show that athletes in general are positively disposed towards injury prevention. However, effective transfer measures are needed to promote use of research evidence in football practice. Future studies should focus on evaluating transfer techniques that influences individual beliefs and attitudes of athletes regarding beneficial prevention strategies.
